# Retraction: Neurological Manifestations of Infectious Diseases: Insights From Recent Cases

**DOI:** 10.7759/cureus.r160

**Published:** 2025-01-16

**Authors:** Jyoti Kashyap, Olusegun A Olanrewaju, Kinza Mahar, Meena Israni, Reena Bai, Narendar Kumar, Komal Kumari, Sujeet Shadmani, Muhammad Arsalan Bashir, Mostafa Elharif, Giustino Varrassi, Satish Kumar, Mahima Khatri, Muhammad Ali Muzammil, Roshan Sharma, Farhan Ullah

**Affiliations:** 1 Medicine, Sri Balaji Action Medical Institute, Delhi, IND; 2 Pure and Applied Biology, Ladoke Akintola University of Technology, Ogbomoso, NGA; 3 General Medicine, Stavropol State Medical University, Stavropol, RUS; 4 Medicine, Bahria University Medical and Dental College, Karachi, PAK; 5 Medicine, Sir Syed College of Medical Sciences, Karachi, PAK; 6 Medicine, Liaquat University of Medical and Health Sciences, Jamshoro, PAK; 7 Internal Medicine, Burjeel Hospital, Abu Dhabi, ARE; 8 Medicine, NMC Royal Family Medical Centre, Abu Dhabi, ARE; 9 Medicine, Shaheed Mohtarma Benazir Bhutto Medical College, Karachi, PAK; 10 Medicine, Ziauddin University, Karachi, PAK; 11 Medicine, Menoufia University, Shibin El Kom, EGY; 12 Pain Medicine, Paolo Procacci Foundation, Rome, ITA; 13 Internal Medicine/Cardiology, Dow University of Health Sciences, Karachi, PAK; 14 Internal Medicine, Dow University of Health Sciences, Karachi, PAK; 15 Medicine, Sanjay Gandhi Memorial Hospital, Delhi, IND; 16 Internal Medicine, Khyber Teaching Hospital, Peshawar, PAK

This article has been retracted by the Editors-in-Chief due to the inclusion of multiple cited sources that do not correspond to the cited text or article topic. The submitting author did not respond to inquiries made by the journal. The journal greatly regrets that this was not identified prior to publication.

